# The Integration of Field Effect Transistors to Microfluidic Devices

**DOI:** 10.3390/mi14040791

**Published:** 2023-03-31

**Authors:** Dhaniella Cristhina de Brito Oliveira, Fernando Henrique Marques Costa, José Alberto Fracassi da Silva

**Affiliations:** 1 Department of Analytical Chemistry, Institute of Chemistry, State University of Campinas (UNICAMP), P.O. Box 6154, Campinas 13083-970, SP, Brazil; 2 National Institute of Science and Technology of Bioanalytics, INCTBio, Campinas, SP, Brazil

**Keywords:** microfluidics, FET, EGFET, bioanalysis, detection

## Abstract

Devices that integrate field effect transistors into microfluidic channels are becoming increasingly promising in the medical, environmental, and food realms, among other applications. The uniqueness of this type of sensor lies in its ability to reduce the background signals existing in the measurements, which interfere in obtaining good limits of detection for the target analyte. This and other advantages intensify the development of selective new sensors and biosensors with coupling configuration. This review work focused on the main advances in the fabrication and application of field effect transistors integrated into microfluidic devices as a way of identifying the potentialities that exist in these systems when used in chemical and biochemical analyses. The emergence of research on integrated sensors is not a recent study, although more recently the progress of these devices is more accentuated. Among the studies that used integrated sensors with electrical and microfluidic parts, those that investigated protein binding interactions seem to be the ones that expanded the most due, among other things, to the possibility of obtaining several physicochemical parameters involved in protein–protein interactions. Studies in this area have a great possibility of advancing innovations in sensors with electrical and microfluidic interfaces in new designs and applications.

## 1. Introduction

The search for sensors that are more sensitive, accessible, and easy to handle has been the subject of different research published in recent years. Such devices must meet parameters, such as high sensitivity and response linearity, in addition to the detection of target analytes in low concentrations or sample amounts [1,2,3]. Among the various types of sensors developed for chemical and biochemical tests, those that use electronic transducers have the largest amount of development given their advantages of low cost and ease of manufacture. Electrochemical sensors based on field effect transistors (FETs) have gained special attention in the development of sensing devices since they present a promising alternative because of their easiness to handle and the wide concentration range [4,5].

Sensors based on FETs are being tested by different groups of researchers and have shown good results in terms of limit of detection, sensitivity, and response time [6,7,8]. Additionally, there is the possibility of miniaturization and free label detection [9]. In view of the aforementioned benefits, FETs have been adapted and used in the development of radiation dose sensors [10,11], glucose [12], oxygen [13], protozoa [14], enzymes [15], hormones [8], and neurotransmitters [16], in addition to biological macromolecules markers of cancers [7] and cardiovascular disease biomarkers [6], among others.

Among sensor research, integrated devices are a promising alternative to reduce high background interference, overcome the complexity of fluid handling, and automate analysis [17,18,19,20,21,22]. These devices can unite different interfaces on the same detection chip. The integration of a semiconductor interface from a FET and channels of a microfluidic chip allows for a broader analysis and the acquisition of more comprehensive physical-chemical parameters, as static and dynamic measurements can be performed.

In this review, we focus on exposing the main advances in the manufacture and application of FETs, especially those extended gate FET types, in sensors for chemical and biochemical analyses. Understanding the current trends in integrated sensors can be helpful for researchers developing other strategies and improvements in sensor technologies.

## 2. Basic Principles Involved in the Operation of Field Effect Transistors

The basic principle of FETs is the modulation of the transport of charge carriers in a semiconductor channel through an external electric field. To understand this phenomenon, it is necessary to understand the structure of FET devices. These are basically constituted by a substrate with three terminals: source, drain, and gate [23]. In them, a conductive channel is generated due to an electrical potential applied to the gate terminal that is positioned parallel to the other terminals. Due to the electrical charge opposite the potential present at the source terminal, the applied charge influences the accumulation of electrically charged species on the surface of the transistor that allow their flow through the substrate [24,25]. This effect is possible due to electron tunneling, which is described by quantum mechanics as the ability of particles to cross high-energy barriers [26]. The tunneling phenomenon contributes to the functioning of the sensor by forming a conductive channel between the source and drain terminals due to the electrons overcoming the energy barrier of the substrate on which they are arranged and connecting directly to each other, reaching a moment when they will not be impediments other than the one initially overcome [26].

Since the emergence of FET devices, several theoretical and experimental studies have been developed, and this has given basis for the emergence of new transistor structures. There is a myriad of terminology for FET structures, including Metal-Oxide-Semiconductor Field-Effect Transistor (MOSFET), Ion-Sensitive Field-Effect Transistor (ISFET), Extended Gate Field-Effect Transistor (EGFET), and Organic Field-Effect Transistor (OFET), which are the most widespread.

MOSFETs are designed from materials with different conductive properties that are arranged interspersed by layers of metals, oxides, and semiconductors. Above a semiconductor layer, which is often made of silicon, an insulating material, commonly silicon dioxide (SiO_2_), is grown via heat treatment. It is responsible for intermediating the charges that will be polarized during the device’s operation. This is one of the main causes of the expansion of silicon (Si) transistors since their emergence. Given their refractory property, they provide high mechanical stability with a lower rate of losses during the manufacture and operation of the device. FETs made of this material have stable and robust manufacturing processes, in addition to low noise and the potential for disposable sensors [27]. Above the insulating layer, a metallic layer is deposited and acts as a contact for the gate voltage [26,27,28]. Lately, some researchers have manufactured gates with polysilicon structures, forming FETs entirely of Si. The advantage of this type of device is related to its efficient consumption of electrical energy, development of cheaper devices, and mass production with reduced distance between the drain/source [29,30,31]. Due to the modulation of the charges on the semiconductor substrate, the oxide, given its high impedance, acts as an insulating layer, causing the current between the drain and source terminals to be controlled by the variation of the voltage signal at the gate, characterizing the field effect [24,25]. The induced channel in the MOSFET can originate from the direct application of potential on the device port or from other sources, such as the deposition of charges in a solution. Thus, it is possible to configure these transistors in different ways for sensing species in a solution, as is the case of ISFETs in which, thinking about the structure of a MOSFET, the metallic gate is removed so that the insulating oxide of the MOS structure is directly exposed to the solution [32]. The membrane in direct contact with the electrolytic solution can contribute to the formation of a double layer at the insulating solution interface, causing the interaction of charges to cause changes in the conductivity of the device channel; thus, the field effect is used in the detection of the ionic potential of the electrolyte from the variation of the sensor threshold voltage [25,26,28,33]. This voltage refers to the moment when the value of the potential between the gate and the source of the device (V_GS_) stimulates the formation of the charge conduction channel in the FET and can be described as:

(1) $$V_{t\left(ISFET\right)}=V_{t\left(MOSFET\right)}+E_{Ref}-\phi_{0}+X^{Sol}-\frac{\phi_{M}}{q}$$

where *V_t(MOSFET)_* is the threshold voltage of the MOSFET, *E_Ref_* is the potential of the reference electrode, *ϕ*_0_ is the surface potential of the electrolyte insulator interface in relation to the volume of the electrolyte, *X^Sol^* is the dipole potential of the electrolyte insulator interface, and *ϕ_M_/q* is the metal work function [34].

In ISFETs, the measurements would occur through direct contact between the gate and the solution, which in some cases can be troublesome. EGFETs have the same response mechanism as ISFETs but, instead of placing the measured solution directly over the gate, a connection is made between the FET and the detection cell [35]. The configuration of an EGFET arises from the connection of a transducer material, such as a sensitive layer to some species, which forms an extension of part of the structure of a MOSFET [25].

EGFETs were proposed in 1983 by Van Der Spiegel et al., in which a selective membrane is deposited in the form of a thin film on a physicochemical transducer that can be connected to a commercial MOSFET or one that is specially designed [36]. The use of commercial MOSFETs makes the manufacturing process of these sensors faster, which contributes to their dissemination. In addition, the extension of the EGFET port allows the device to be used in a greater number of measurements, as the port can be easily replaced. In these devices, the variation of electrochemical potential between the electrolyte and the insulator of the sensitive membrane is given by the charge concentration, which leads to modulation of the gate potential, modifying the resistance of the channel between the source and the drain of the MOSFET, thus affecting the drain-source current (I_DS_). Moreover, EGFET sensors feature a simple structure and easy packaging process with greater stability because only their sensitive part is in direct contact with the solution [35].

Among the works published in the area of integrated sensors, EGFETs have been the most used type of design for integration into microfluidic chips. This is exclusively due to its advantage of insulating the gate electrode, which makes the measurement results more reliable. In addition, the EGFET has a sensing structure that can be easily removed, allowing the use allows multiple uses of the sensor with the possibility of integrating the gate electrode in the most varied types of microfluidic structures. Using new designs and surface engineering with the optimization of different parameters, mainly related to the sensing membrane composition, means that these devices have been explored in different detections.

In research on EGFETs, a major noticeable obstacle that boosted the integrated use of this in microfluidic chip is related to the difficulties in measurements that have strong background electronic signals coming from the electrical response of other species present in the analyte.

OFETs differ from other FETs in that instead of using a metal as a gate electrode, a conductive polymer or other organic material with conductive properties is used [37]. Their advantages are that they provide multivariate measurements with good sensitivity and specificity, real-time operation, and remarkable stability [38]. The limitations related to these are the fact that stable organic materials with high electronic mobility are still very scarce, restricting the development of this type of sensor [39].

In making devices with electrical and microfluidic interfaces, both principles involved in the isolated operation of these devices are combined and explored. This allows more information about the sample to be collected, making the coupling an indispensable tool in some applications, especially those involving a very small volume of sample, as is the case of studies on cellular activities.

## 3. Field Effect-Based Sensors Integrated into Microfluidic Chips: Current Progress

### 3.1. Integrated Systems for Simultaneous Detections and Point of Care

**Figure 1:**
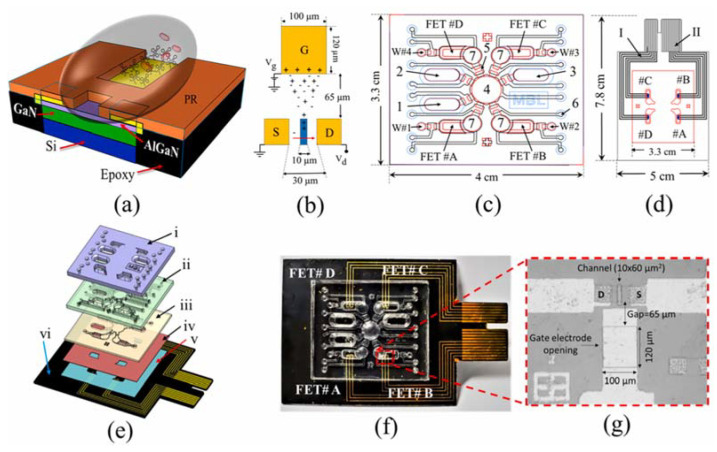
Integrated sensor design. (**a**) Side view of the sensor showing semiconductor packaging and materials (PR = photoresist). (**b**) Design of the source (S), drain (D), and gate (G) sensor terminals. Vg = gate voltage and Vd = drain voltage. (**c**) Microfluidic chip with the three reservoirs (1; 2; 3) to store the samples and reagents needed for detection, (4) micropump, (W1, 2, 3, and 4) waste collection chambers, (5) micropump fluid contact, (6) air inlet, and (#7) micromixers. Four field effect transistors were used. (**d**) Shows the position of the FET sensor arrays. (**e**) Layers of the microfluidic chip: top reagent reservoir (i), pneumatic control layer (ii), liquid flow channel layer (iii), thin PDMS layer (iv), double-sided tape (v), and FET-embedded epoxy substrate (vi). (**f**) Photograph of the integrated sensor. (**g**) Zoom view of the FET area. Reprint by permission from Elsevier.

The possibility of using the same integrated system to carry out simultaneous detections arouses the interest of several research groups. Initiatives in this sense allow the monitoring of multi-species in a small sample volume and fluid load detection. In the work of Sinha et al. [18], a chip composed of four FETs in contact with microfluidic channels distributed in the same structure was developed. The system was portable and had an automated process that required little reagent and sample. It was used in the simultaneous sensing of four cardiovascular disease biomarkers that were identified by interactions with self-assembled monolayers of aptamers immobilized on a gold surface. Figure 1 depicts the proposed multi-sensor.

The epoxy substrate was adhered to on which the FETs were distributed, and it served for the physical contact of the electrodes with the solution. The chip channels had dimensions of 10 × 60 µm^2^ with a 100 µm × 120 µm sensor port. As the device was automated, it also had reservoirs for storing samples and washing solutions, in addition to micropumps and waste reservoirs.

The sensor was applied for the recognition of cardiac troponin I (cTnI) biomarkers, N-terminal pro b-type natriuretic peptide (NT proBNP), fibrinogen, and C-reactive protein (CRP) from predefined aptameric sequences for each molecule separately. The interaction between the species and the aptamers causes variations in the gate potential, which alters the drain-to-source current (I_DS_). In this way, changes in the capacitance of the solution modulated the drain current of the FET. The sensor had a response time of five minutes and a limit of detection (LOD) of 0.14 mg L^−1^, 0.832 pg mL^−1^, 0.394 pg mL^−1^, and 20.2 mg dL^−1^ for CRP, NT-proBNP, cTnI, and fibrinogen, respectively. The recognition of cTnI and NT proBNP biomarkers surpassed those acquired via ELISA (Enzyme-Linked ImmunoSorbent Assay) assays. This work was the first to report the application of a sensor that allowed the simultaneous detection of four biomarkers in a single clinical sample and that did not require human intervention during the process in a portable device. Monitoring more than one biomarker species reduces lengthy laboratory tests, materials, and leading time for results and allows for better clinical decision-making.

**Figure 2:**
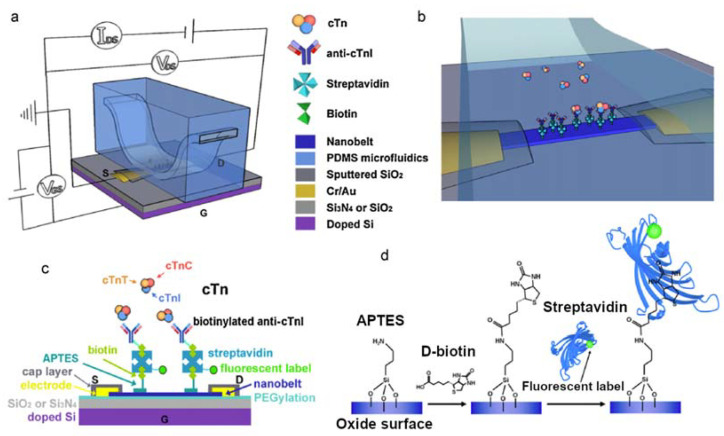
(**a**) Representation of the integrated chip and the electrical connections used in the study. S = source, D = drain, and G = gate. (**b**) Simplified model of cTnI binding in the microfluidic channel. (**c**) Detailed schematic of the detection system assembly. (**d**) Schematic diagram of the surface biotininization of the nanobelt of SnO_2_. Reprint by permission from Elsevier.

Cheng et al. [40] also developed an integrated sensor for the detection of cardiac troponin I, but in their study, they prepared a membrane functionalized with nanobelts of SnO_2_ (tin dioxide). The nanobelts were grown by physical vapor deposition and their use in the sensor provided that the surface-to-volume ratio was maximized. The surface of the nanobelts was silanized with aminopropyltriethoxysilane (APTES) to obtain a multilayer of silanols that served as a binding surface for D-biotin molecules. These molecules acted as streptavidin receptors that were identified by dual detection with the electrical response coming from the FET and an optical response given by the use of a fluorescence microscope. The microfluidic channel was made in PDMS and had a figure U geometry with the sensitive area positioned in the lower contact of the structure supporting the chip. Figure 2 summarizes the operation of the device made by the authors.

In the first tests with the sensor, the authors modified the reaction medium in different pH ranges to verify how the device behaved. They observed that the sensor response was dependent on the pH of the sample medium due to the isoelectric point of streptavidin and changes in the net charge of the molecule. After verifying that the sensor was able to identify proteins, it was used in the device for the recognition of cTnI within the human cardiac troponin complex and they obtained good selectivity in the species. The antigen–antibody binding that occurred showed a sensitivity of 100 pM for cTnI (~2 ng/mL), supporting the use of the sensor for these detection purposes.

The work presented above brings a new material, SnO_2_. It composes the class of binary oxides, and such materials are useful due to their small size, ease of processing, high carrier mobility, compatibility with CMOS TFT (Thin Film Transistors), and transparent CMOS processes. They have multifunctionality and a high dielectric constant, in addition to high thermodynamic and kinetic stability when in contact with the silicon base [41]. Research in this direction has invested in the use of different oxide materials other than those based on Si to obtain a better accumulation of induced charges due to greater electrical conductivity, stability, and capacitance.

The works presented above solve a relevant scientific gap related to the manufacturing of chips for the detection of cardiovascular diseases. Investments in this field of study can contribute to the reduction of annual mortality rates from diseases caused by heart damage, which are equivalent to 31% of annual deaths recorded by the World Health Organization (WHO). Multispecies detections allow a broader diagnosis of the patient’s life situation and, when performed on the same sample, reduce costs and waste from tests.

Point of care (POC) devices have become widespread since they globalize the use of sensors, allowing a wide group of people to access this technology. In a study by Song et al. [20], a POC device with a new sensor design was presented. It was based on the new strategies of wrapped nanotechnology with a 3D profile. In the research, coiled nanoFETs conjugated by layers of indium nitride, silicon nitride, and silicon oxide were integrated, which formed a structure of InN/SiN_x_/SiO_x_ on a microfluidic chip. This work was the first to explore the properties of InN for the formation of microtubes, betting on the high electron density of the surface of this material, which was the main advantage of the study. In addition, the rolled nanotechnology of the material was only possible due to the property of elastic tension, which must be thought of when we consider the manufacturing of chips, such as the one reported here. However, the main disadvantage when we need to use this type of material is the laborious preparation process that requires appropriate machinery and consequently results in the final value of the device [42,43]. The sensor structure was used in the detection of antibodies related to the human immunodeficiency virus—HIV—in order to enable faster clinical results.

**Figure 3:**
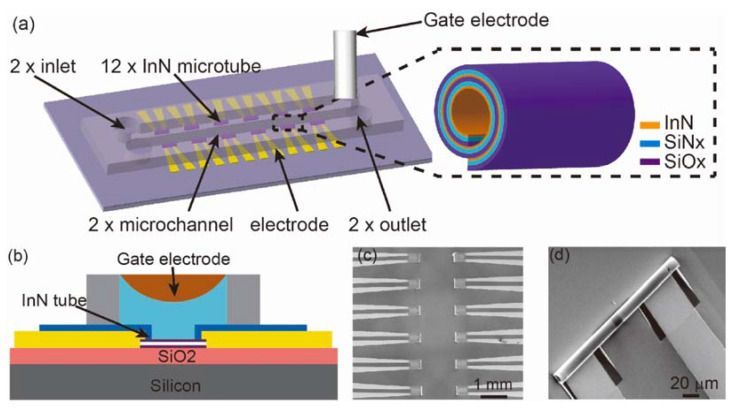
(**a**) FETs distributed along two microfluidic channels and (**b**) the FET architecture. (**c**) Scanning electron microscopy (SEM) of the sensor array. (**d**) Magnification of the SEM image with an emphasis on the structure of the InN microtube. Reprint by permission from Elsevier.

The chip was formed by two microfluidic channels 400 µm wide and 6.5 mm long, with the sensing part distributed along the two channels. The InN/SiN_x_/SiO_x_ microtubes were 350 µm in length and 20 µm in diameter, and they were distributed at 650 µm spacing along the channel. This allowed placing 12 microtubes 20 µm in length along the flow channels. In the experimental condition, a single gate electrode was used to perform the measurements (Figure 3).

For the fabrication of the devices, different techniques of deposition of the layers were carried out, including reactive direct current (DC) spraying for the InN film and plasma-enhanced chemical vapor deposition (PECVD) for the layers of SiN_x_/SiO_x_. The 3D structure of the microtubes naturally formed microfluidic ducts where samples could flow and was arranged in two channels on the chip, with six FETs responsible for each channel. Using 3D technology, it was possible to manufacture sensors in batches. This is one of the important differentials of this research, in addition to the use of the same-shared gate electrode for all microtubes.

In the study, the authors theoretically determined the chip geometries from the concave surface of the InN to obtain the reduction of possible experimental interferences, such as electrostatic shielding. They explored the high electron density on the surface of InN to assess its influence on sensitivity to HIV-1 gp41 antibodies diluted in phosphate-buffered saline (PBS). The antibodies bound to APTES immobilized on the surface of the InN. The sensor response came from the charge change on the surface of the InN/SiN_x_/SiO_x_ microtubes when there was the presence of the species of interest. In this way, detection was performed and a LOD of 1 ng mL^−1^ was reached.

With the advent of the SARS-CoV-2 pandemic, different strategies for the detection of this virus were developed and among them, the exploration of the potential of integrated systems with the use of FETs has also been registered. In the research by Gao et al. [44], the authors fabricated an in situ structure for the detection of the SARS-CoV-2 virus from the stacking π–π of graphene oxide and graphene, in which antigen antibodies modulated the gate potential and gave the sensor response. This was the first work to develop a carbon allotrope heterostructure FET biosensor for the detection of SARS-CoV-2.

In the study, the microfluidic chip was designed in polydimethylsiloxane (PDMS) with channels in which graphene oxide and then graphene were inserted until they interacted and formed the heterostructure. Once formed inside the microfluidic channel, capture antibodies were added and interacted with the heterostructure, preparing the sensitive membrane of the sensor. The heterostructure had its conductivity improved due to the opening of the band gap by the variation in the hybridization of the material. This allowed for better payload mobility and increased sensor sensitivity. Despite the good results reported in this research, the fabrication of graphene sensors is not compatible with the conventional microfabrication processes of thin film transistors, which gives is its main disadvantage. Its use, however, overcomes the disadvantage of the non-transparency of silicon substrates, which makes it possible to obtain better cellular images [45].

The integrated biosensor developed was tested in a bovine serum albumin buffer solution that was enriched with the virus and carried out detection in a unit of 1 μL of the sample with a LOD of up to 8 fg mL^−1^ of SARS-CoV-2 protein in a response time of just 20 min. The authors compared the results obtained with those predicted by the use of graphene FET (Gr-FET) and realized that the designed device had a sensitivity three times greater than what they attributed to graphene stacking. As for stability, it was also improved by the heterojunction due to the network defects of the materials being compensated. The research made it possible to detect the presence of the virus quickly and in the early stages, which could help with more timely treatment and better control of this viral infection.

The research by Mandal et al. [46] proposed the development of a prototype for the detection of a prostate-specific antigen (PSA), a marker of prostate cancer. The study sought to overcome the unsatisfactory detection limits obtained with labeled POCs. The difference was that the sensor was based on dielectrophoresis and labels, accessible on a disk microfluidic platform. The system required the use of coplanar gate electrodes and the FET was formed from graphene. The system was the first label-free system to detect PSA and the results were completely satisfactory with a detection limit of 1pg/mL when the target was placed in human serum. Usually, the order of nanograms determines the screening of patients so, in the range acquired with the sensor, it was possible to identify the formation of the disease in an early stage [46].

The latest research reported here used graphene transistors. This material has a transparency of 98% and is ultrathin and ultralight, with a Young’s modulus of 1.0 TPa, an intrinsic resistance of 130 Gpa, a planar density of 0.77 mg m^−2^, a conductivity thermal power of 8000 W m^−1^ K^−1^, a conductivity of 1.42 × 10^6^ S m^−1^, a resistance of 125 Ω sq^−1^, and an electronic mobility of 2 × 10^5^ cm^2^ V^−1^ s^−1^, among other properties that make its use attractive, mainly because it is the material with the highest conductive capacity at room temperature. Although it has good properties, a fact that should be considered corresponds to the use of this platform for the immobilization of probes, such as DNA, due to instabilities reported in the literature that demonstrated that this immobilization can affect the conductivity of graphene [47,48].

Due to the possibility of graphene suffering, device performance degradation has given the zero band gap and the indeterminate drift of the Dirac point, and some research has adhered to the use of molybdenum disulfide (MoS_2_) in different geometries. This material has low noise, is easy to manufacture, low cost, and has a lower leakage current compared to graphene. It also have good LOD and sensitivity, in addition to low throughput, and it suffers less from integrity problems [49].

**Figure 4:**
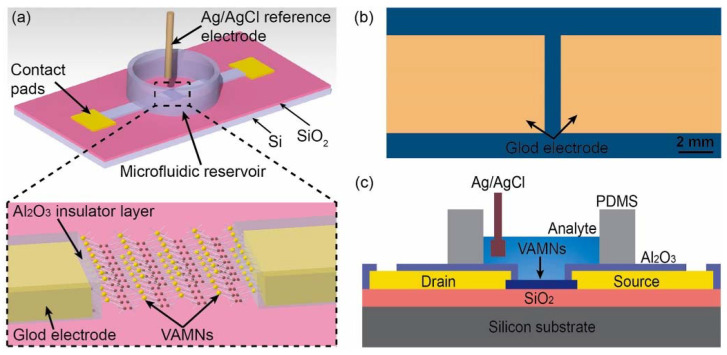
(**a**) Structure formation zoom for design visualization VAMNS = MoS_2_ monolayers aligned vertically. (**b**) VAMNS structure. (**c**) Experiment settings. Reprint by permission from Elsevier.

Song et al. [50] prepared vertically-aligned MoS_2_ nanolayers (VAMNs), structures to explore contributions in band gap transitions given the number of layers. The great challenge of this type of structure is the immobilization of capture probes in the basal plane of the MoS and the reproducibility of these planes. The thickness of the channel created and the separation generated between molecules and targets are important points to be considered when working with 2D MoS. To overcome the problem of immobilization, the authors used nanolayers of material aligned vertically, as shown in the image below (Figure 4).

The device detected label-free PSA and obtained an LOD of 800 fg mL^−1^, which was compared to the 8 pg mL^−1^ obtained in ELISA assays. Manufactured in batches on Si substrate, they used the Sputtering method to coat the Si wafer plus oxide with the Mo film. The immobilization of the biomolecules occurred by Van der Waals force in the 2D molecular layers by the use of surface reagents. The degradation of the carrier mobility (on/off) and the decrease in the FET channel conductance of the system were explained by the thin dielectric layer that directly induced the VAMN potential and degraded the channel mobility and conductance. This effect also occurred due to the local geometric deformation. Depending on the pH, the PSA charge could change and this led to the presence of an unwanted charge in the measurement due to potential blocking effects.

The advantage reported in the study is linked to the easy standardization of the channels. The reported sensor is the only one that obtained such a sensor configuration with application results better than the ELISA assay standards. The study is a major advance for research on label-free immunosensors.

#### 3.1.1. Indium Gallium-Zinc Oxide Transistors Integrated into Microfluidic Chips

EGFETs based on materials other than silicon are also being used in the manufacturing of integrated sensors. These attribute new conductive properties to the sensor, making measurements more efficient.

A material that has stood out among research on sensors is the indium gallium-zinc oxide (IGZO) transistors [51,52]. This material has conductive properties superior to silicon substrates, presenting low processing temperature, high electrical mobility, and non-toxicity, in addition to the simplicity of functionalization on surfaces [38]. Additionally, this material does not require a doping process, since the oxygen vacancies existing in it, which are inherent to its structure, can act as electron donor species. The main advantage when we think of IGZO is its low energy consumption and better optical transparency than Si, but it can have the disadvantage of high defects during manufacturing, which causes problems in the stability of the films.

**Figure 5:**
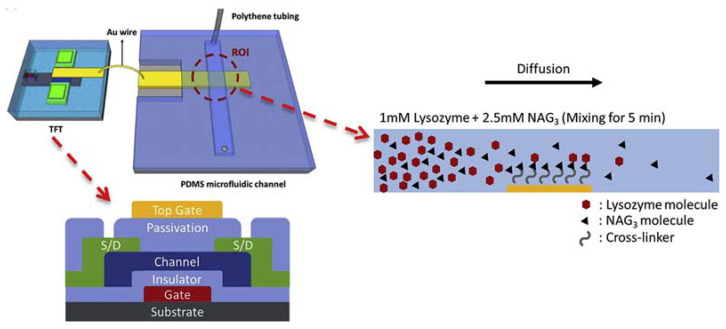
Schematic diagram of the integrated sensor with an extended gate and a representation of the biomolecule interaction. Reprint by permission from Elsevier.

Chou et al. [21] integrated thin-film transistors (TFT) of IGZO into a microfluidic chip forming a biosensor that was used in real-time studies of protein binding kinetics. The approach used by the authors did not require labeling or immobilization of target proteins on the detection surface; instead, a dynamic flow analysis was performed. Using a device configuration (Figure 5) where the FET port was divided into two structures that could be loosely fitted together, they were able to extract information about the variations in the drain current and consequently calculate the association and dissociation rates involved in the interaction of lysozyme with its ligand tri-N-acetyl-D-glucosamine (NAG 3).

The detection of molecules occurred as follows. In the microfluidic channel, the proteins underwent diffusion and the film of the double-gate thin-film transistor transduced the biological signal in a variation of the drain current due to the induced transient current in the solution. This was possible because while the lower port acted as a kind of reference electrode, charges were induced in the upper port of the sensor. In the study, dissociation constants (K_d_) were monitored in mixing ratios of proteins of 1:2.5; 2.5:2.5, and 2.5:1, resulting in K_d_ values of 44.02, 50.84, and 83.35 μM for each proportion, respectively. Data obtained from protein interaction studies, such as the one developed by the authors, are of paramount importance to assist in drug development, mainly because they allow a better understanding of the kinetics involved in the process of drug identification by the receptor.

**Figure 6:**
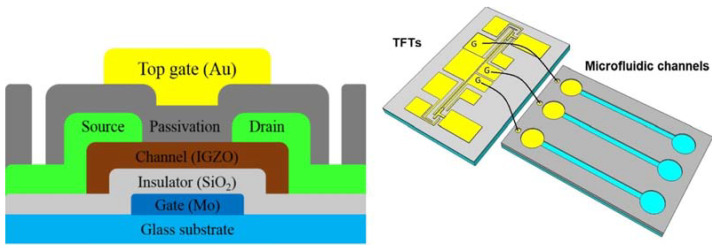
On the (**left**), the cross-sectional view of the FET sensor and on the (**right**), the integrated system formed by a microfluidic chip with three channels and an integrated circuit containing three *n*-type MOSFETs. Reprint by permission from Elsevier.

A similar study was developed by Chen et al. [22], where they also used an IGZO transistor but focused on the study of the transient analysis of the streptavidin-biotin complex. The channels were designed on a glass substrate that was external to the sensor, thus preventing contamination and allowing for multiple uses of the device. The integrated system was formed by a microfluidic chip with three channels and an integrated circuit containing three *n*-type MOSFETs (Figure 6).

The mechanism of response is based on the modulation of the drain source current (I_DS_) caused by the complex formation between streptavidin and biotin, which alters the gate potential. Using this method, the authors obtained information about the different electrical and diffusion properties of the proteins and also of the complex, resulting in that streptavidin, due to its larger molecular radius, presented a longer diffusion time than biotin, regardless of the concentration ranges. When the complex was formed, due to its larger size, its diffusion coefficient was smaller, which made it circulate at a lower speed through the microfluidic channel. To validate the data obtained by the electrical measurements, the authors also performed optical tests using a fluorescence microscope.

**Figure 7:**
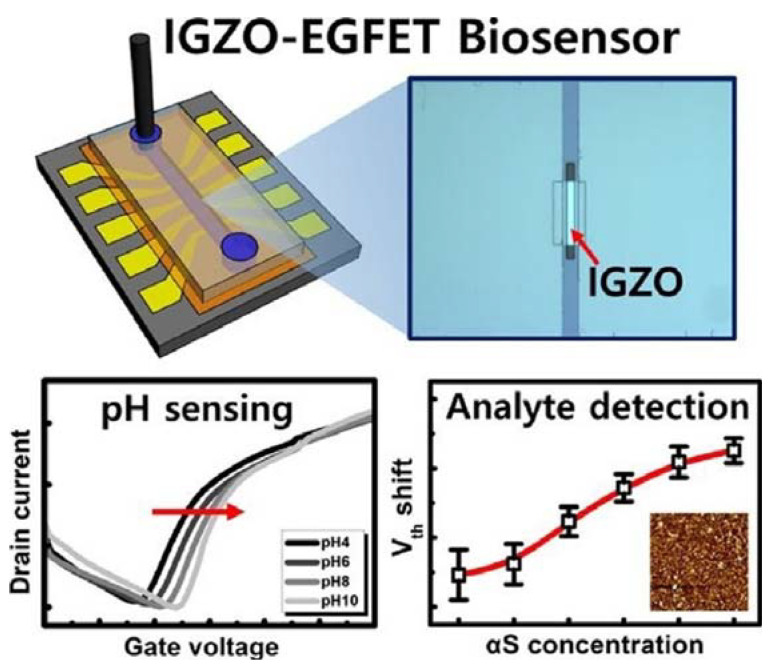
Top—schematic structure of the integrated system. Bottom—variation of the characteristic response as a function of the pH (**left**) and threshold voltage (V_th_) as a function of α-synuclein (αS) concentration (**right**). Reprint by permission from Elsevier.

In the study by Chae et al. [53], the authors developed an IGZO-based EGFET and evaluated the feasibility of the designed sensor in biological sensing. For this purpose, the authors deposited IGZO films via sputtering and radiofrequency that were used as the electrical channel and biological interface of the sensor. The authors prepared the membrane structure from surface modifications of IGZO with APTES that received monoclonal antibody SPM451 for binding to α-synuclein proteins, a marker of Parkinson’s disease. In the study, the authors prepared devices with the modification by the reagent and others without the modification for comparative purposes. They also miniaturized the active channel of the device so that the sensor operates at low power and manufactured the system in batches to compare its parameters and check for possible divergences in efficiency values. The thin IGZO film acted as an active electrical layer and was 80 µm^2^ wide and 10 µm^2^ long. With these measurements, the authors obtained 32 devices using 4 inches of substrate. Each device had five individual active channels, as represented in Figure 7.

In batch manufacturing, the devices had a coefficient of variation of 11.4%, which is within the acceptable range. When the sensor acted in the pH recognition, the sensitivity of the modified sensor was twice as high as the one that was not modified. In the protein detection, the sensor worked in a concentration range of 10 fg mL^−1^ to 1 ng mL^−1^ with a linearity of 9.35 mV decade^−1^, proving to be a good example of manufacturing a sensor in situ as well as a promising candidate in clinical analyzes involving the α-synuclein protein.

#### 3.1.2. Integrated Flexible/Wearable FETs

Recently, the development of flexible sensors with the possibility of adaptations for wearable devices that are efficient in monitoring species in body fluids other than blood has received important attention. In this sense, non-invasive biosensors using saliva, sweat, and tears are expanding our understanding of the distribution of relevant species in biological fluids and their advantages are, among others, their low weight and low operating voltage.

In the work by Laliberte et al. [19], the authors reported the development of a wearable chip that couples graphene field effect transistors, GFETs, in a microfluidic structure for the detection of interleukin—6 (IL-6), a protein that acts in the acute inflammatory response of the human body. The GFETs, due to their formation in graphene, had two-dimensional geometry with malleable mechanical properties. In the authors’ study, these properties were explored in the construction of the sensor with the graphene surface modified by aptamers. The aptamers promoted the formation of antigen–antibody-like interactions that modulate the gate potential and, consequently, the conductance of the FET.

The device manufactured by the authors had a battery-powered circuit that was used for sensor control and data recording. This system made it possible to explore the autonomy of the biosensor and POC applications. By using the device, they obtained a detection range of 10 pM to 100 nM, which is compatible with other studies already published. Moreover, the device showed good functionality even with mechanical modifications in the structure, at least between a radius of curvature of 1.5 cm to 4.25 cm. Designing standalone devices reduces the possibility of human interference in measurements and allows for easier sensor manipulation.

Graphene-based devices have been widely used in protein detection research as the basis for active layers, mainly those formed by DNA probes [54]. As initially addressed in Section 3.1, graphene-based devices suffer from the choice of the graphene transfer method that can affect the electrical characteristics of the material, causing changes in the Dirac point. Transfer processes involve different conditions and times, depending on the procedure, and damage or doping effects may occur on the graphene surface—factors that can alter the properties of the materials. The Dirac point voltage given the bipolarity of graphene is used for the detection of the target analyte. The recognition mechanism occurs when ions accumulate in the active layer on the graphene surface, changing the channel conductance. This conductance can be associated with the target concentration in the sample and thus it is possible to carry out detections in different concentration ranges [55].

#### 3.1.3. Organic FETs

FET devices that have organic films are called organic transistors and their integration into microfluidic chips is also reported in the literature. In the study by Koklu et al. [56], a glucose detection system was developed using an organic electrochemical transistor (OECT). They used polymers for structure development and functionalized the gate electrode with glucose oxidase enzymes that helped with glucose recognition. The work was the first to integrate a microfluidic system into an OECT operating in accumulation mode for detection using enzymes and in real-time.

The OECT was developed on a glass substrate by using a photolithography process and showed increased sensitivity and stability, which are essential for portable and autonomous devices. The electrolyte concentration controlled the OECT supply voltage and the existing channel between the source and drain was structured from the organic conductor. The sensor was the first to be reported in the literature to allow glucose detection without the need for an additional amperometric detector for H_2_O_2_ monitoring. Compared to detection results from other chips, such as those made in PEDOT:PSS, the manufactured sensor had a higher sensitivity. Despite the good results, when integrated into the channels of a microfluidic chip, the stability and sensitivity obtained did not increase, and only the signal-to-noise ratio improved.

Ricci et al. [57] used an EGOFET to detect the a-Synuclein protein, indicative of degenerative diseases such as Parkinson’s and Alzheimer’s. The aim of the study was the detection of Parkinson’s and an LOD of 0.25 pM was achieved. The sensor was label-free by exploiting the organic semiconductor channel for signal transduction with different strategies for surface functionalization with anti(a-Synuclein).

The difference between OECTs and an EGOFET is in the mode of operation. While OECTs exploit both diffusion and electrochemical doping of the detector film, EGOFETs depend on the capacitance between the gate electrode and the active material [57]. Overall, the advantage of using organic FETs is that they are ultrasensitive and highly specific for bioanalytical assays. They have greater proximity to the aqueous medium and the possibility of interface or integration with matrices with living systems. Its disadvantage corresponds to the materials. There are few stable organic materials with high electronic mobility, thus restricting the development of this type of sensor [39,57].

### 3.2. Devices with Non-Electrical Counterparts

To reduce the interference of inconvenient species to the sensitivity of the sensor, in the research by Kim et al. [58], the authors developed a biosensor based on EGFETs with the extended gate of the device formed in the bottom area of the microfluidic channel. This configuration surpassed the planarity of this type of transistor and was used in the detection of the streptavidin-biotin complex. The authors used a gold electrode as a gate terminal with a self-assembled monolayer rich in thiol groups. The authors also performed tests with a Surface Plasmon Resonance (SPR) biosensor to compare the results and validate the strategy used in the research.

The detection principle used in this study was like that in the study by Cheng et al. [40], in which the formation of the complex modified the charge coming from the functional groups of the molecules and modulated the sensor current.

**Figure 8:**
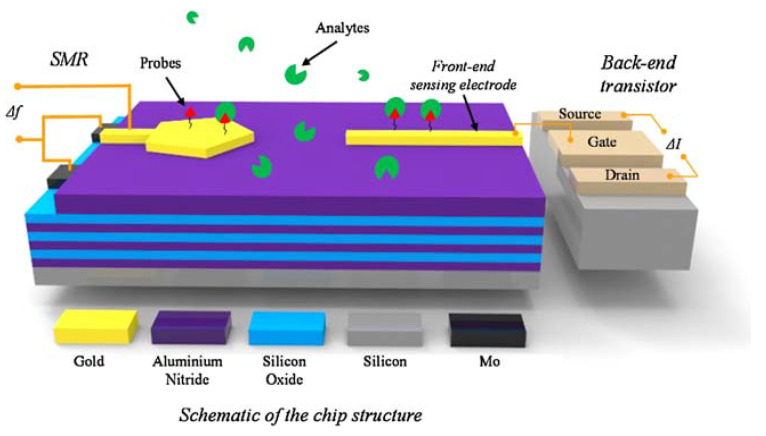
System used in the dynamic analysis of the interaction between anti-PSA and native human PSA. Reprint by permission from Elsevier.

Another study on integrated systems that deserves to be mentioned was the one developed by Liu et al. [59], who performed a static and dynamic analysis. The sensor had, in addition to the EGFET, solidly mounted resonators (SMRs) and allowed for a multiplexed analysis to detect molecular interactions and obtain different physical-chemical parameters involved in the interactions. In the system, shown in Figure 8, the measurements were independent, while the FETs were responsible for the static analysis. The resonators obtained data from the dynamic analysis of the interaction between the anti-human prostate specific mouse monoclonal antigen (anti-PSA) and the native human prostate antigen (PSA) with its respective antibody pairs.

In the study, the authors used a matrix containing four pairs of extended gates made of Au, and the principle involved in the detection via resonators occurred due to the exposure of the sensor to an electric field that was responsible for generating acoustic energy within the piezoelectric layer of the SMR. When the species of interest was present in the sample, the solution flow modified the mechanical vibration of the piezoelectric layer according to the present concentration.

In the study by Park et al. [60], silicon FET structures were manufactured for dual electrochemical and fluorescence detection. As the authors aimed for electrical and optical detection, the device required a good level of transparency for better image resolution. For this, it was necessary that the sealing of the liquid in the channel was thin and they bet on the sensor design. Named Trench FET, the device containing four FETs in a *p*-type substrate allowed for monitoring the movement of charged materials, including DNA, on a hybrid PDMS/glass microfluidic channel. The great innovation of the study was that it did not have a metallic gate contact since the charged target was responsible for the potential modulation. The authors also manufactured a sensor with a metallic gate for comparative purposes. The fluorescence for optical detection came from carboxylate-modified fluorescent microspheres. Although the sensor obtained interesting results, it required preamplifiers to apply drain voltage and detect current IDs. The bimodal measurement was also not effective for the simultaneous use of the sensor since the photoelectric current caused noise in the electrical measurement.

**Figure 9:**
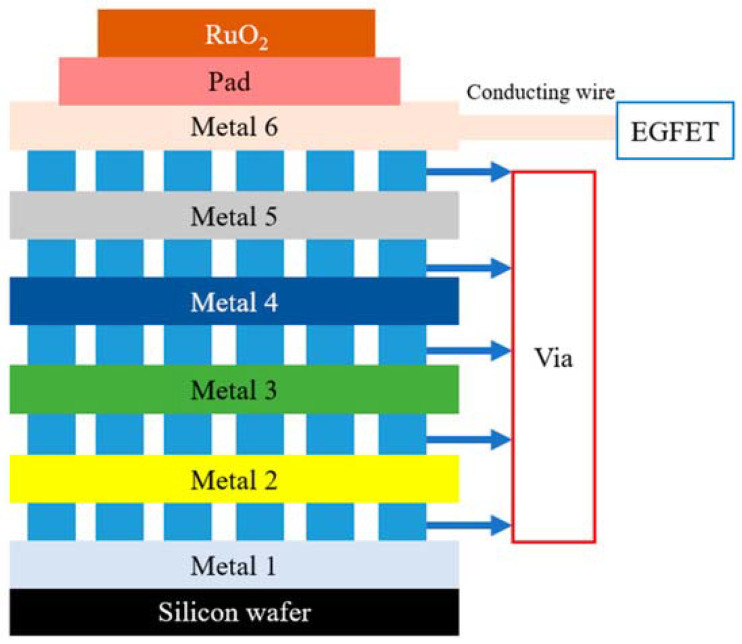
Fabricated device structure. Pad = connection pad.

Other models that have also emerged are those with sequential detection windows, as reported by Kuo et al. [61]. In this study, a device was made up of six aluminum metallic structures to prevent a measurement with only one recognition structure from collapsing the probe port and damaging the sensor (Figure 9).

The authors carried out static and dynamic measurements in which the last access metal transmitted the gate response signal of a commercial MOSFET through the contact pads (Pad). The detection was due to the surface potential of the binary oxide (RuO_2_) varying with the lactic acid concentration. With a gate voltage of 1.8 V, the sensitivity and linearity under static conditions were 61.62 mV mM^−1^ and 0.991, respectively. The values of these same parameters when under dynamic conditions were 81.31 mV mM^−1^ of sensitivity and 0.995 of linearity at a flow of 30 µL min^−1^.

### 3.3. Chloride Detection Devices

In the research by Chou et al. [62], a new EGFET device format was used, where an extended gate was integrated into a microfluidic chip aiming for chloride detection. The microfluidic platform was designed by photolithography and etching technology and the EGFET had two ports, an upper one composed of a membrane of chloride ions and a lower port that was formed by a film of ruthenium dioxide (RuO_2_) bonded to ions of chloride. The device was tested in sodium chloride (NaCl) solutions at concentrations from 1 M to 10^−5^ M under static and dynamic conditions. As result, it was found that the sensor had better sensitivity and linearity for NaCl concentrations in the range of 10^−1^ M and 10^−4^ M. Under dynamic conditions, the sensitivity and linearity of the sensor were better than under static conditions. This changed when flow rates greater than 15 μL min^−1^ were applied. The system had a detection limit of 10^−4^ M, a linearity range from 0.1 to 10^−4^ M, the best flow rate at 15 μL min^−1^, a mean sensitivity of 33.73 mV decade^−1^, and a linearity of 0.996.

In another study by the same group, Tseng et al. [63] again used thin films made of RuO_2_ to investigate the dynamic detection characteristics with different flow rates (5 μL min^−1^–40 μL min^−1^) for chloride ion recognition but in this case, the RuO_2_ film was enriched with graphene oxide (GO) in different proportions. The authors tested two EGFET configurations, one structured as PET/RuO_2_/GO/chloride ion and the other without GO, and the results obtained proved that the use of GO increased the detection surface area and, consequently, an increase in the sensitivity of the sensor to chloride ions. The best results were obtained at a flow rate of 30 μL min^−1^ with a sensor sensitivity equal to 66.673 mV decade^−1^, a linearity of 0.998, and a ratio of RuO_2_/GO of 0.010% (*w/w*). The data obtained in this new study were again superior to the static measurements, demonstrating the potential of a dynamic microfluidic measurement.

We saw earlier that the detection method in EGFETs is based on the site-binding model and the electrical double-layer theory. Considering these models for a pH measurement configuration, their functioning can be explained by a protonation/deprotonation reaction occurring in the sensor membrane, which is responsible for controlling the surface potential and providing a net charge. This is an important detection principle used in EGFET sensors that occurs not only in the identification of protons but expands to other species and, due to this, it can be used in different detections. This possibility meant that these sensors were designed for other measurements, such as the one mentioned above.

### 3.4. Other Applications

With the emergence of new applications of materials for FET devices, processes for obtaining Si were modified to find out if this material could also have optical properties, for example. Then, Si-porous devices appear to have electrical and optical functionalities [64,65].

In the research by Ahmed et al. [66], EGFETs based on Si-porous manufactured in a sheet of Si *n*-type of crystallographic orientation 111 were used. The device was used to monitor the hydrogen potential of the medium.

Ma et al. [67] developed an integrated chip for the detection of bacteria involved in the manifestation of tuberculosis. For this, the authors built a multichannel microfluidic structure in which 360 ultrasensitive Si-based nanowire transistors were integrated, which served as a sensor film for the capture probe, and electrochemical recognition occurred due to the antigen–antibody binding.

## 4. Conclusions

In this article, we reviewed the main advances of integrated sensors, focusing on the potential use of field effect transistors in the development of devices that allow the performance of chemical and biochemical analyses. Based on the research results presented here, we see that devices with engineered interfaces will be the key to the next generation of sensors. These interfaces can encompass different transducer materials such as optical, electromechanical, resistive, capacitive, etc., for the development of simultaneous and autonomous detection systems. In sensors that require an electrical interface, EGFETs seem to be a good alternative for the construction of molecular recognition platforms because when they are integrated into microfluidic chips, they allow measurements at low sample levels and a decrease in the background signal, in addition to presenting significant results in static and dynamic analyses.

In the research presented in this article, those that used EGFET-type FETs also showed lower detection limits, which can be linked to the structure of this type of transistor with better encapsulation and flexibility of the gate terminal for integration into more complex microfluidic platforms. In general, clinical diagnosis, mainly, can be increasingly improved with the use of these sensors and with the development of new ones, opening a range of possibilities for research in this area, which has been a fertile field for new discoveries.

## Data Availability

Not applicable.
